# Moderate Hypothermia Provides Better Protection of the Intestinal Barrier than Deep Hypothermia during Circulatory Arrest in a Piglet Model: A Microdialysis Study

**DOI:** 10.1371/journal.pone.0163684

**Published:** 2016-09-29

**Authors:** Mengya Liang, Kangni Feng, Xiao Yang, Guangxian Chen, Zhixian Tang, Weibin Lin, Jian Rong, Zhongkai Wu

**Affiliations:** 1 The Second Department of Cardiac Surgery, the First Affiliated Hospital, Sun Yat-Sen University, Guangzhou, China; 2 Assisted Circulatory Laboratory of Health Ministry, Sun Yat-sen University, Guangzhou, China; 3 Department of Cardiopulmonary Bypass, the First Affiliated Hospital, Sun Yat-Sen University, Guangzhou, China; Azienda Ospedaliero Universitaria Careggi, ITALY

## Abstract

**Introduction:**

This study aimed to assess the effects of different temperature settings of hypothermic circulatory arrest (HCA) on intestinal barrier function in a piglet model.

**Methods:**

Twenty Wuzhishan piglets were randomly assigned to 40 min of HCA at 18°C (DHCA group, n = 5), 40 min of HCA at 24°C (MHCA group, n = 5), normothermic cardiopulmonary bypass (CPB group, n = 5) or sham operation (SO group, n = 5). Serum D-lactate (SDL) and lipopolysaccharide (LPS) levels were determined. Microdialysis parameters (glucose, lactate, pyruvate and glycerol) in the intestinal dialysate were measured. After 180 min of reperfusion, intestinal samples were harvested for real-time polymerase chain reaction and western blotting measurements for E-cadherin and Claudin-1.

**Results:**

Higher levels of SDL and LPS were detected in the DHCA group than in the MHCA group (P < 0.001). Both MHCA and DHCA groups exhibited lower glucose levels, higher lactate and glycerol levels and a higher lactate to pyruvate (L/P) ratio compared with the CPB group (p<0.05); the DHCA group had higher lactate and glycerol levels and a higher L/P ratio (p<0.05) but similar glucose levels compared to the MHCA group. No significant differences in E-cadherin mRNA or protein levels were noted. Upregulation of claudin-1 mRNA levels was detected in both the DHCA and MHCA animals’ intestines (P < 0.01), but only the DHCA group exhibited a decrease in claudin-1 protein expression (P < 0.01).

**Conclusion:**

HCA altered the energy metabolism and expression of epithelial junctions in the intestine. Moderate hypothermia (24°C) was less detrimental to the markers of normal functioning of the intestinal barrier than deep hypothermia (18°C).

## Introduction

Although the use of hypothermic circulatory arrest (HCA) improves the surgical outcomes of complex cardiac surgery, it is still associated with relatively high mortality and complication rates[1,2]. It is estimated that endotoxemia following HCA surgery due to malfunction of the intestinal mucosal barrier affects postoperative recovery in up to 60% of critical patients and may even be associated with multi-organ failure [3]. Impairment of the intestinal mucosal barrier and increased intestinal wall permeability have been observed during normothermic cardiopulmonary bypass (CPB), which would result in rapid metabolic deterioration within the intestinal wall and translocation of luminal bacteria into the systemic circulation[4]. The effects of HCA on the brain and heart have been studied thoroughly, and a small number of previous studies have investigated several aspects of bowel function from histological morphology to microcirculatory blood flow during HCA [5,6]. However, due to the absence of generally accepted and precise monitoring techniques, few data were available concerning real-time metabolic changes within the intestine in the presence of HCA[7,8]. Nonetheless, cell junctions in the intestinal epithelium play vital roles in maintaining the integrity and normal function of the intestinal barrier, and alterative expression of cell junctions was observed in animal models of acute intestinal ischemia[9]. However, whether and how HCA causes changes in the expression of intestinal epithelial junctions is yet to be identified. In the present study, piglet models with two HCA temperature settings (18°C HCA and 24°C HCA) were used to investigate whether and how the interstitial metabolic parameters and the expression of cell junctions change in the intestine after HCA. Additionally, based on these results, a comparative analysis was made to evaluate which temperature setting was most beneficial for preserving intestinal function during the HCA procedure.

## Methods and Materials

### Animal care and experimental protocol

Twenty Wuzhishan piglets (Suibei Nursery of Laboratory Animals, Guangzhou, China) of either sex (6 to 8 weeks of age and weighing 9.6 ± 2.4 kg) were randomly assigned to four groups: (1) the deep hypothermic circulatory arrest group (DHCA group, n = 5), which received HCA at 18°C for 40 min; (2) the moderate hypothermic circulatory arrest group (MHCA group, n = 5), which received HCA at 24°C for 40 min; (3) the cardiopulmonary bypass group (CPB group, n = 5), which underwent normothermic cardiac arrest with cardiopulmonary bypass for 40 min; and (4) the sham operation group (SO group, n = 5). All procedures performed on the animals were approved by the Institutional Animal Care Committee of Sun Yat-sen University and conducted in compliance with Guidelines for Animal Experimentation of Sun Yat-sen University.

### Perioperative management

As previously described [10], conditioned animals were anesthetized with an intramuscular administration of 40 mg/kg ketamine and 2 μg/kg fentanyl for induction and were intubated and mechanically ventilated. Anesthesia was maintained via intravenous infusion of fentanyl, vecuronium bromide and midazolam. An arterial pressure catheter was inserted via the right femoral artery for pressure monitoring and blood sampling. Hemodynamic parameters and rectal temperature were recorded with a multichannel physiologic recorder (MP150, BIOPAC Systems, California, USA).

### Management of cardiopulmonary bypass and HCA

As previously described [10], the extracorporeal circuit was primed with blood from another piglet. CPB flow was maintained between 75 and 80 ml·kg^-1^·min^-1^, which corresponded to a systemic perfusion pressure between 50 and 80 mmHg. The desired body temperature was maintained using a heat exchanger (Sarns, Ann Arbor, MI, USA).

**Fig 1:**
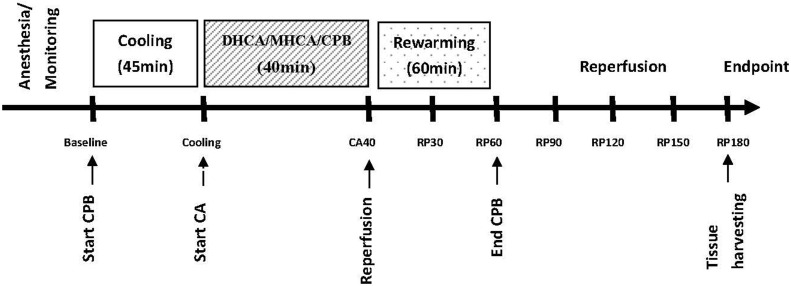
Experimental protocol. CA40, circulatory arrest for 40 min; RP30, reperfusion for 30 min; RP60, reperfusion for 60 min; RP 90, reperfusion for 90 min; RP 120, reperfusion for 120 min; RP 150, reperfusion for 150 min; and RP 180, reperfusion for 180 min.

The animals were cooled systemically for at least 40 min. The ascending aorta was cross-clamped, and cardioplegic solution was administered when rectal temperature reached 32°C. For the MHCA and DHCA groups, circulation was ceased for 40 min after the target temperature (18°C or 24°C) was reached. Then, the cross-clamp was removed and the animal was rewarmed to normothermia using a temperature gradient of 8°C. When the hemodynamics stabilized, the animals were weaned from CPB. Reperfusion continued for 3 h. Only the SO group animals underwent mid-sternal thoracotomy and were anesthetized until specimen preparation. The experimental protocol is shown in Fig 1.

### Intestinal microdialysis

After anesthesia, a transrectus incision was performed, thereby allowing access to the peritoneal cavity. A microdialysis catheter (CMA70, Microdialysis AB, Sweden) was implanted in the intestinal wall of the jejunum 20 cm distal to the Treitz ligament. The catheter was perfused with Ringer’s solution in situ for 45 min before measurements, and a constant flow at 2 μl/min was maintained using a microdialysis pump (CMA106, Microdialysis AB, Sweden). Samples were analyzed immediately on-site for glycerol, lactate, pyruvate and glucose concentrations using enzymatic fluorometric assays (CMA 600 Microdialysis Analyzer, Microdialysis AB, Sweden). The in vitro recovery rate was 63% for lactate, 44% for pyruvate, 35% for glycerol and 35% for glucose.

### Blood sampling and specimen preparation

Blood samples were collected at each time point in the experimental protocol. Serum D-lactate levels were measured using an enzymatic spectrophotometric assay. Serum lipopolysaccharide (LPS) levels were determined using a modified perchloric acid extraction. Animals were euthanized after 3 h of reperfusion, and samples of the jejunum 10 cm distal to the Treitz ligament were harvested. The intestinal mucosa from the specimen was gently scraped off and preserved at -70°C for subsequent real-time PCR and western blot assessments.

### Real-time PCR

Total RNA was extracted from the collected intestinal mucus using Trizol reagent (Sangon, Shanghai, China). First-strand cDNA was synthesized from total RNA using the SYBR PrimeScript^™^ RT-PCR Kit (Takara, Dalian, China) with six random primers and an oligo(dT) primer. The polymerase chain reactions (PCRs) were performed in 96-microwell plates in 20-μl reaction volumes. Relative transcript abundance was determined using the LightCycler480 software (Roche, Switzerland) according to the 2-ΔΔCt method. β-actin amplification signals were employed as internal controls. Three replicates were performed per sample, and negative controls with no template were included.

### Western blot

The intestinal samples were mixed with phosphate saline buffer (pH 7.4) containing 1 mmol/l PMSF and 1 mmol/l EDTA-free complete protease inhibitor and then homogenized and centrifuged at 4°C 12,000 rpm for 20 min. The supernatant fluid was collected and electrophoresed on sodium dodecyl sulfate–polyacrylamide gels, transferred to a polyvinylidene difluoride membrane (Immobilon; Millipore, Billerica, MA, USA), and then probed with the anti-E-cadherin antibody (Abcam, Cambridge, MA, USA) and anti-claudin-1 antibody (Invitrogen, Carlsbad, CA, USA). Signals were detected with an enhanced chemiluminescence system (Millipore, Billerica, MA, USA).

### Statistical analysis

Statistical analyses were performed using SPSS 20.0 software for Windows. Data for continuous variables are expressed as the mean ± standard deviation ($\bar{x}\;\pm \;SD$). ANOVA for repeated measurement was used to evaluate significant differences in continuous variables among groups at different time points, and paired comparison of each two groups was performed with Bonferroni adjustment. P < 0.05 was defined as statistically significant.

## Results

### Histological morphology

**Fig 2:**
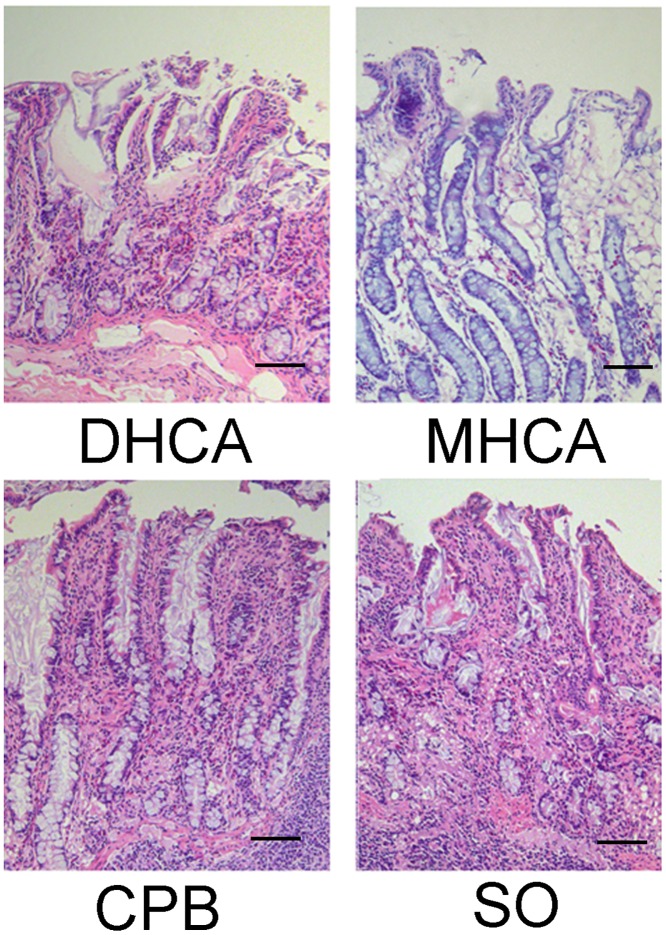
Hematoxylin-eosin staining of the intestinal tissue. The mucosal villi and glands were normal in the intestinal epithelia of the SO and CPB groups. Marked edema of the mucosal villi, increased gaps between the epithelial cells and inflammatory cell infiltration were observed in the MHCA and DHCA groups. Magnification: 100 folds; the scale bars indicate 100 μm.

Small intestine segments were stained with hematoxylin-eosin. As shown in Fig 2, marked edema of the mucosal villi, increased gaps between the epithelial cells and inflammatory cell infiltration were observed in the MHCA and DHCA groups.

Histological changes of the intestinal mucosa were evaluated independently by two pathologists who were blinded to the study groups using the modified Chiu’s method[11]. The Chiu scores of the DHCA and MHCA groups were significantly higher than those of the SO and CPB groups (*P* < 0.001). There was no significant difference between the SO and CPB groups (*P* > 0.05), and no significant difference was noted between the MHCA and DHCA groups (*P* > 0.05; Table 1).

**Table 1:** Chiu’s scores based on the intestinal hematoxylin-eosin staining (mean ± sd).

	Group			
	DHCA(n = 5)	MHCA(n = 5)	CPB(n = 5)	SO(n = 5)
Chiu’s Score	2.9 ± 0.5*	2.6 ± 0.5*	0.4 ± 0.5	0.5 ± 0.5

* P <0.001 for both DHCA and MHCA groups compared with the CPB and SO groups, respectively. SO, sham operation group; CPB, cardiopulmonary bypass group; DHCA, deep hypothermic circulatory

### Serum D-Lactate and Lipopolysaccharide

**Fig 3:**
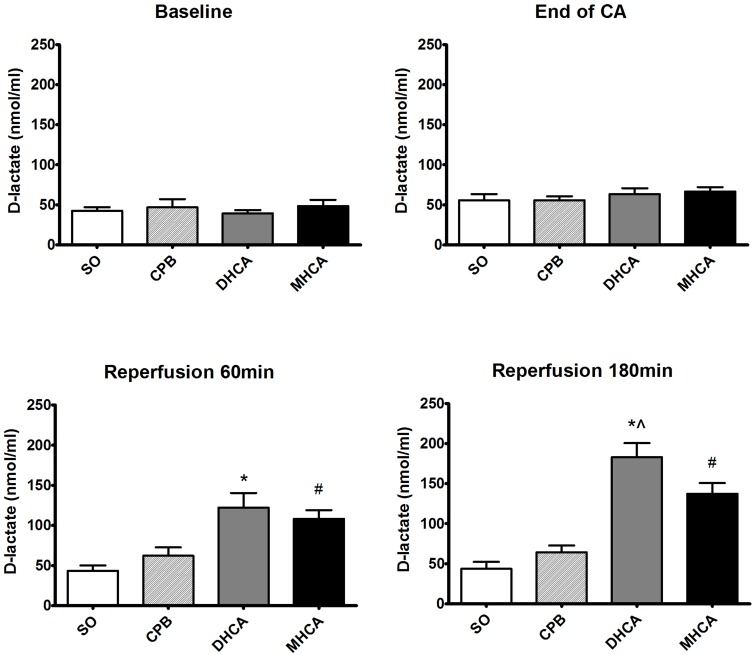
Serum D-lactate levels at 180 min of reperfusion. SO, sham operation group; CPB, cardiopulmonary bypass group; DHCA, deep hypothermic circulatory arrest group; MHCA, moderate hypothermic circulatory arrest group. Mean±SD. n = 5. *P < 0.001 DHCA vs. CPB (t = 7.330 at 60 min of reperfusion and t = 15.32 at 180 min of reperfusion); # P < 0.001 MHCA vs. CPB (t = 6.898 at 60 min of reperfusion and t = 9.415 at 180 min of reperfusion); and ^ P < 0.05 DHCA vs. MHCA (t = 5.515 at 180 min of reperfusion).

**Fig 4:**
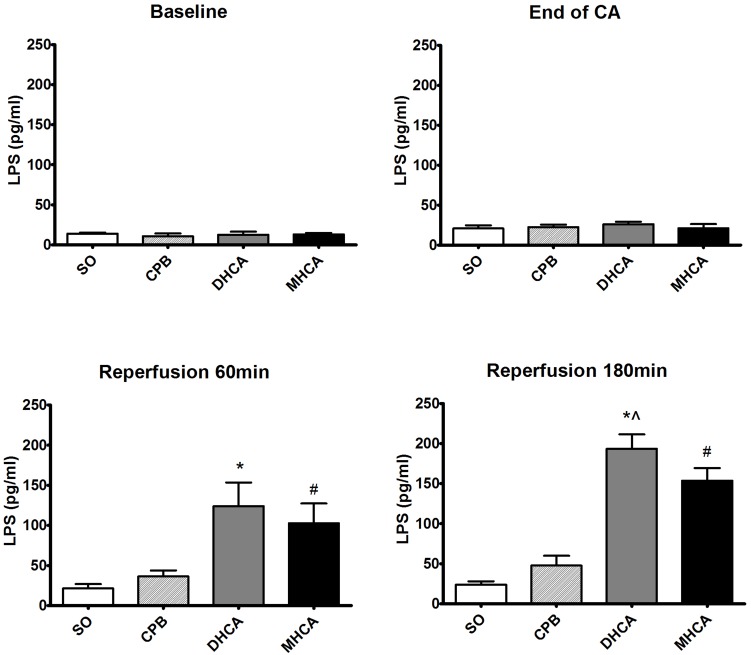
Serum lipopolysaccharide (LPS) levels at 180 min of reperfusion. SO, sham operation group; CPB, cardiopulmonary bypass group; DHCA, deep hypothermic circulatory arrest group; MHCA, moderate hypothermic circulatory arrest group. Mean±SD; n = 5. *P < 0.001 DHCA vs. CPB (t = 8.414 at 60 min of reperfusion and t = 18.710 at 180 min of reperfusion); # P < 0.001 MHCA vs. CPB (t = 6.363 at 60 min of reperfusion and t = 13.601 at 180 min of reperfusion); and ^ P < 0.05 DHCA vs. MHCA (t = 5.105 at 180 min of reperfusion).

The serum D-lactate and LPS levels are presented in Figs 3 and 4. At 60 min and 180 min of reperfusion, both the DHCA and MHCA groups exhibited significantly higher levels of D-lactate compared to the CPB group (p<0.001). Only at 180 min of reperfusion was a difference in D-lactate levels between DHCA and MHCA animals noted (t = 5.515, p<0.001). As shown in Fig 4, both the DHCA and MHCA groups exhibited higher levels of LPS than the CPB group at 60 min and 180 min of reperfusion (p<0.001). At 180 min of reperfusion, the LPS level was found to be higher in the DHCA group than in the MHCA group (t = 5.105, p<0.001).

### Intestinal microdialysis parameters

**Fig 5:**
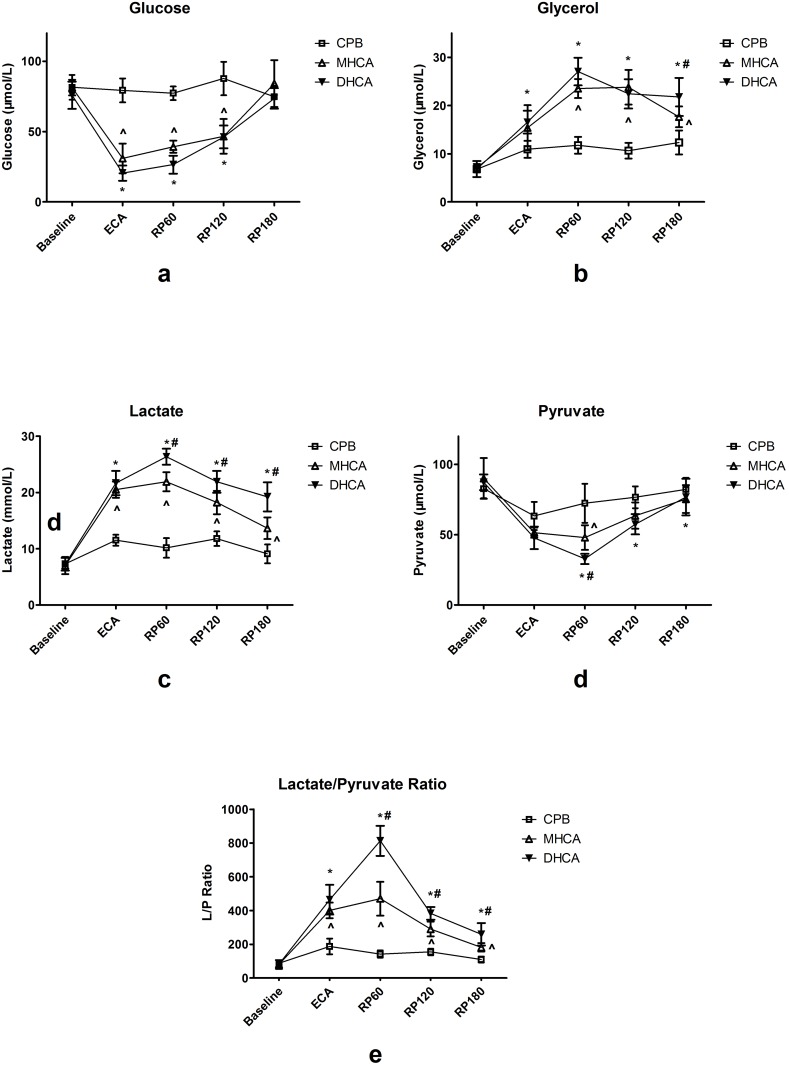
**a-e. Microdialysis parameters in interstitial fluid of small intestine.** Fig 5a, Glucose; Fig 5b, Glycerol; Fig 5c, Lactate; Fig 5d, Pyruvate; Fig 6e, Lactate/ Pyruvate ratio. CPB, cardiopulmonary bypass group; DHCA, deep hypothermic circulatory arrest group; MHCA, moderate hypothermic circulatory arrest group. ^ P<0.05 MHCA vs CPB; * P<0.05 DHCA vs CPB; # P<0.05 DHCA vs MHCA. ECA, end of cardiac arrest; RP60, reperfusion for 60min; RP120, reperfusion for 120min; RP180, reperfusion for 180min.

Real-time measurements of intestinal metabolic parameters are presented in Fig 5. No difference was noted at baseline among the groups (all P>0.05).

Except for the CPB group, glucose levels in the interstitial space of the intestinal wall decreased in both the MHCA and DHCA groups during CA and then increased following reperfusion. The DHCA group had a lower glucose level than the MHCA group during both CA and the early reperfusion phase (ECA, RP60 and RP120 time points, P < 0.05) (Fig 5a).

Glycerol levels were elevated in the two HCA groups after reperfusion, remained at higher levels compared with the CPB group, and failed to normalize, even at the end of the study (P < 0.001). At 180 min following reperfusion, the glycerol levels of the DHCA group were significantly higher than those of the MHCA group (Fig 5b).

As demonstrated in Fig 5c, the lactate levels in both the MHCA and DHCA groups raised to a significantly higher level compared to those of the CPB group (P < 0.001) after the initiation of CA. Furthermore, the DHCA group exhibited higher lactate levels throughout the reperfusion phase than the MHCA group (P < 0.05).

As presented in Fig 5d, pyruvate levels in all groups were reduced after CA. In the reperfusion phase, the pyruvate levels in the DHCA and MHCA groups remained at a significantly lower level compared with those of the CPB group (P < 0.05). A more reliable parameter of metabolic disorder, the L/P ratio, showed that the two HCA group exhibited more severe metabolism disturbance than the CPB group during and after CA (P < 0.001); moreover, the DHCA group’s L/P ratios were significantly higher than those of the MHCA group throughout the reperfusion phase (P < 0.001) (Fig 5e).

### *E-cadherin* and *claudin-1* mRNA expression

**Fig 6:**
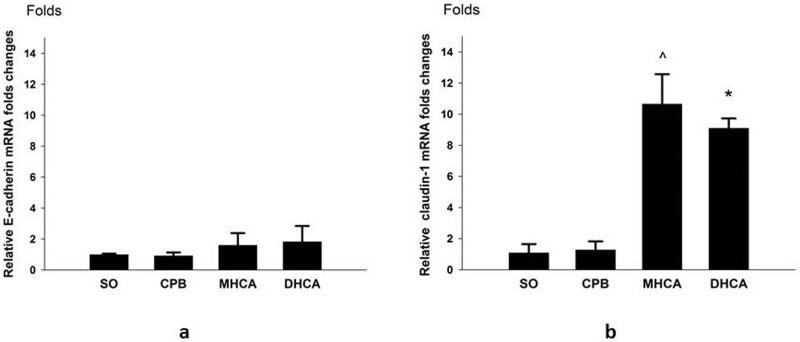
**a-b. e-cadherin and claudin-1 mRNA levels determined by real-time PCR.** (a) e-cadherin mRNA levels at 180 min of reperfusion. The relative fold changes of e-cadherin in the SO, CPB, MHCA and DHCA groups were 1.001 ± 0.057, 0.925 ± 0.207, 1.607 ± 0.775 and 1.837 ± 1.002, respectively. (b) claudin-1 mRNA levels at 180 min of reperfusion. The relative fold changes of claudin-1 in the SO, CPB, MHCA and DHCA groups were 1.092 ± 0.555, 1.291 ± 0.535, 10.664 ± 1.903 and 9.106 ± 0.623, respectively. Mean±SD; n = 5. ^P < 0.05 DHCA vs. CPB; * P < 0.05 MHCA vs. CPB.

The *e-cadherin* and *claudin-1* mRNA levels determined by real-time PCR are shown in Fig 6. No significant differences in *e-cadherin* mRNA levels were noted between the four groups (all *P* > 0.05; Fig 6a). As shown in Fig 6b, the *claudin-1* mRNA levels of the DHCA group were significantly higher than those of the CPB and SO groups at 180 min of reperfusion (*P* < 0.01). Additionally, a significantly higher *claudin-1* mRNA level was observed in the MHCA group at 180 min of reperfusion compared to that in the CPB and SO groups. *Claudin-1* mRNA expression did not differ significantly between the DHCA and MHCA groups (*P* > 0.05).

### E-cadherin and Claudin-1 protein levels

**Fig 7:**
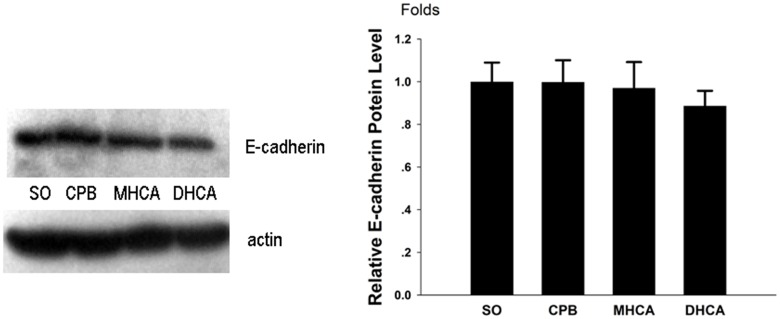
E-cadherin protein expression levels determined by western blot. The relative fold changes of E-cadherin in the SO, CPB, MHCA and DHCA groups were 1.000 ± 0.090, 0.998 ± 0.103, 0.971 ± 0.121 and 0.886 ± 0.071, respectively. Mean ± SD; n = 5.

**Fig 8:**
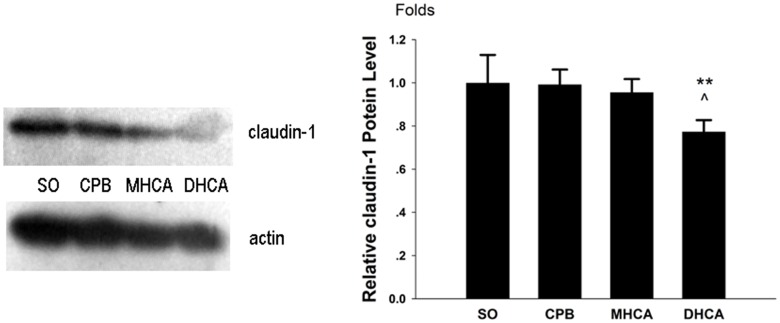
Claudin-1 protein expression levels determined by western blot. The relative folds changes of Claudin-1 in the SO, CPB, MHCA and DHCA groups were 1.000 ± 0.130, 0.992 ± 0.069, 0.956 ± 0.062 and 0.784 ± 0.054, respectively. Mean ± SD; n = 5. **P < 0.05 DHCA vs. CPB; ^ P < 0.05 DHCA vs. MHCA.

As demonstrated in Figs 7 and 8, similar to the mRNA expression patterns, the expression patterns of the two tight junction proteins in the intestinal mucosal tissues were different. The E-cadherin protein expression exhibited no significant differences between the groups (*P* > 0.05; Fig 7). However, compared with the other three groups, a significantly lower level of Claudin-1 protein was detected in the DHCA animals’ intestines at 180 min of reperfusion (*P* < 0.01; Fig 8).

## Discussion

Currently, due to the wide application of HCA in cardiac surgery, intestinal barrier impairment has become a frequent complication following HCA in critical patients[8,9,11]. Although hypothermia has well-established protective effects against ischemia/reperfusion injury for most organs[12], its intestinal protection efficacy is still unknown and controversy remains regarding the optimal temperature to utilize during HCA. One probable reason for these ongoing questions is that until now there were no generally accepted and precise monitoring parameters available for evaluating the effects of different temperatures. The microdialysis technique is capable of detecting real-time metabolic changes in vivo, which makes it a sensitive and site-specific method for monitoring real-time metabolic changes within the intestine in the presence of HCA[13]. Additionally, investigating the expression of cell junctions provides a novel perspective on the exacerbation of intestinal injury due to their importance in preserving normal function of the mucosal barrier. The current study is the first to demonstrate the real-time metabolic changes and expression of cell junctions in the intestine during HCA.

As demonstrated by the histological results, severe damage to the intestinal mucosal epithelium due to HCA was observed. Additionally, destruction of the intestinal barrier frequently occurs following HCA and results in increased intestinal permeability. D-lactate is naturally produced by gut bacteria, and serum LPS level has been shown to be a useful marker for monitoring intestinal permeability and bacterial translocation [14]. Markedly elevated serum D-lactate and LPS levels were detected in the reperfusion phases, indicating that substantial impairment of the intestinal barrier and increased intestinal permeability did occur during HCA.

As a result of the intestinal mucosal barrier impairment and subsequent increased permeability, substantial variations in microdialysis parameters within the interstitial space of the intestine were observed in the two HCA groups. (1) Glucose level in the perfusate is closely correlated with perfusion and energy supply to the intestine[15]. In the present study, decreased glucose was observed immediately after CA in DHCA and MHCA animals, indicating that the intestinal mucosa was depleted of its energy supply as a result of perfuse cessation. (2) Elevation of glycerol is usually interpreted as a biomarker of lipid hydrolysis and cell membrane destruction[16]. Undoubtedly, our results suggest that destruction of the cell membrane due to HCA occurred in the reperfusion phase. In particular, a significant difference between the DHCA and MHCA groups was found at 180 min of reperfusion, indicating that 24°C provided better preservation of cell membrane integrity than 18°C (3). Under index ischemia, cellular metabolism shifts from aerobic to anaerobic glycolysis, thereby resulting in the release and accumulation of lactate[17]. The lactate level change pattern in the DHCA and MHCA groups demonstrated the deterioration of metabolism in the intestine, which was synchronous with the glucose level variation. It can be inferred that animals with 24°C HCA had a better metabolic state within the intestine than those with 18°C HCA. (4) The L/P ratio is a more reliable parameter for characterizing the relationship between aerobic and anaerobic metabolism [18]. The animals in the DHCA group showed rapid depletion of energy supply to the intestine with an immediate increase of the L/P ratio and a peak value at 60 min following reperfusion. The L/P ratio curve of the MHCA group formed a flat slope lower than that of the DHCA group, indicating that 24°C HCA caused milder energy disorders in the intestine than 18°C HCA.

Cell junctions play key roles in the maintenance of normal intestinal function. Tight junctions (TJs) and adhesion junctions (AJs) are the two most important categories of cell junctions for maintaining intestinal barrier integrity [14]. Claudins and E-cadherin are the main components of TJs and AJs, respectively. Alternation in the expression patterns of these junctions drastically increases paracellular permeability and is fundamental to many physiological processes [19,20]. To our knowledge, this study is the first to demonstrate the effects of HCA on the expression of intestinal cell junctions. Interestingly, the results indicated that their expression patterns were different. Both the mRNA and protein expression patterns of Claudin-1 were remarkably affected by HCA, whereas those of E-cadherin were not. In the animals treated with HCA, decreased Claudin-1 protein expression and an upregulation of mRNA level were observed. Based on the trends in the mRNA and protein expression variations, it can be inferred that increased transcription of *claudin-1* represented a compensatory response to the diminution of Claudin-1 protein expression. One possible reason for the lack of alteration in E-cadherin expression lies in its intestinal distribution pattern [21]. TJs form the major paracellular barrier that separates the apical and basolateral compartments of membranes; TJs are mostly found on the luminal side of the intestinal apical junctional complex, whereas AJs form a continuous belt between the epithelial cells and are more likely located near the basal membrane [20,22]. It can be inferred from these results that the expression of claudin in the intestinal tract is more sensitive than E-cadherin to microenvironmental changes during HCA.

Although hypothermia has well-established protective effects against I/R injury for most organs, including the intestine [12], not all low temperatures are beneficial. Excessive hypothermia causes irreversible organ damage [23]. The appropriate temperature to utilize during the HCA procedure is one of the major concerns regarding the safety of this operation. Some previous studies demonstrated that milder injuries to the intestinal barrier occur at 20°C compared to 30°C after gut ischemic insults [24]; however, the correlation between temperature and intestinal function has seldom been investigated[25,26]. In the present study, two temperature levels were evaluated. Despite similar morphological changes demonstrated by Chui’s scores, 24°C HCA exhibited better intestinal protective effects than 18°C HCA based on the findings of lower serum D-lactate and LPS levels. For the first time, we provided real-time metabolic data regarding intestinal mucosa at different temperatures during HCA by microdialysis: 24°C HCA had lower levels of lactate, glycerol and L/P ratio than 18°C HCA, suggesting that intestinal metabolism is less suppressed during moderate hypothermia than during deep hypothermia. Additionally, this model demonstrated how temperature affected the expression of intestinal epithelial cell junctions. Neither the protein nor the mRNA levels of E-cadherin exhibited any difference between these two temperature settings. Meanwhile, Claudin-1 protein expression was lower with 18°C HCA compared with 24°C HCA, while the mRNA levels did not differ. This disparity was most likely due to the following factors: (1) 18°C caused more severe injury to the structure of intestinal barrier [22], as demonstrated by the results of decreased claudin-1 expression. (2) Although genetic transcription for epithelial regeneration recovered in both groups immediately after reperfusion initiation, the lower temperature and longer rewarming time required obviously hindered protein synthesis in the DHCA group. In summary, these above-mentioned results suggested that 24°C HCA is more beneficial than 18°C HCA for preserving the normal function of the intestinal epithelial barrier.

Several limitations of the present study should be noted: [1] the animals were euthanized immediately at the end of the procedure, which limited any investigation of long-term outcomes; [2] the widely used test markers of gut permeability (e.g., 3-O-methyl-D-glucose, D-xylose or lactulose), which are able to access the intestinal barrier function more intuitively, were not employed in the current study; and [3] the expression patterns of the other categories of cell junctions, including gap junctions and desmosomes, were not investigated in the current study and thus require further research.

## Conclusion

This study was the first one to investigate the effects of HCA on intestinal metabolism and the expression of cell junctions. The results demonstrated that HCA did have a significant effect on the energy metabolism and expression of epithelial junctions in the intestine. It can be inferred that moderate hypothermia (24°C) was less detrimental to the markers of normal functioning of the intestinal barrier than deep hypothermia (18°C).

## Supporting Information

S1 Fig Claudin-1 protein expression levels determined by western blot (the first blot). This figure shows the original uncropped blots of Claudin-1 at 180 min following reperfusion. SO, sham operation group; CPB, cardiopulmonary bypass group; DHCA, deep hypothermic circulatory arrest group; MHCA, moderate hypothermic circulatory arrest group.
(TIF)

S2 Fig Claudin-1 protein expression levels determined by western blot (the second blot). This figure shows the the original uncropped blots of Claudin-1 at 180 min following reperfusion. SO, sham operation group; CPB, cardiopulmonary bypass group; DHCA, deep hypothermic circulatory arrest group; MHCA, moderate hypothermic circulatory arrest group.
(TIF)

S3 Fig E-cadherin protein expression levels determined by western blot (the first blot). This figure shows the original uncropped blots of E-cadherin at 180 min following reperfusion. SO, sham operation group; CPB, cardiopulmonary bypass group; DHCA, deep hypothermic circulatory arrest group; MHCA, moderate hypothermic circulatory arrest group.
(TIF)

S4 Fig E-cadherin protein expression levels determined by western blot (the second blot). This figure shows the original uncropped blots of E-cadherin at 180 min following reperfusion. SO, sham operation group; CPB, cardiopulmonary bypass group; DHCA, deep hypothermic circulatory arrest group; MHCA, moderate hypothermic circulatory arrest group.
(TIF)

S5 Fig Molecular size markers GAPDH by western blot. This figure shows the original uncropped blots of molecular size markers GAPDH.
(TIF)

S6 Fig Claudin-1/GAPDH ratio. This figure shows the Claudin-1/GAPDH ratio of the groups. SO, sham operation group; CPB, cardiopulmonary bypass group; DHCA, deep hypothermic circulatory arrest group; MHCA, moderate hypothermic circulatory arrest group. *P < 0.05 DHCA vs. CPB
(TIF)

S7 Fig E-cadherin /GAPDH ratio. This figure shows the E-cadherin/GAPDH ratio of the groups. SO, sham operation group; CPB, cardiopulmonary bypass group; DHCA, deep hypothermic circulatory arrest group; MHCA, moderate hypothermic circulatory arrest group.
(TIF)
