# Supplementary figures and images for: Moderate Hypothermia Provides Better Protection of the Intestinal Barrier than Deep Hypothermia during Circulatory Arrest in a Piglet Model: A Microdialysis Study
Source: PLoS One. 2016 Sep 29;11(9):e0163684. doi: 10.1371/journal.pone.0163684 (PMC5042434; doi:10.1371/journal.pone.0163684)

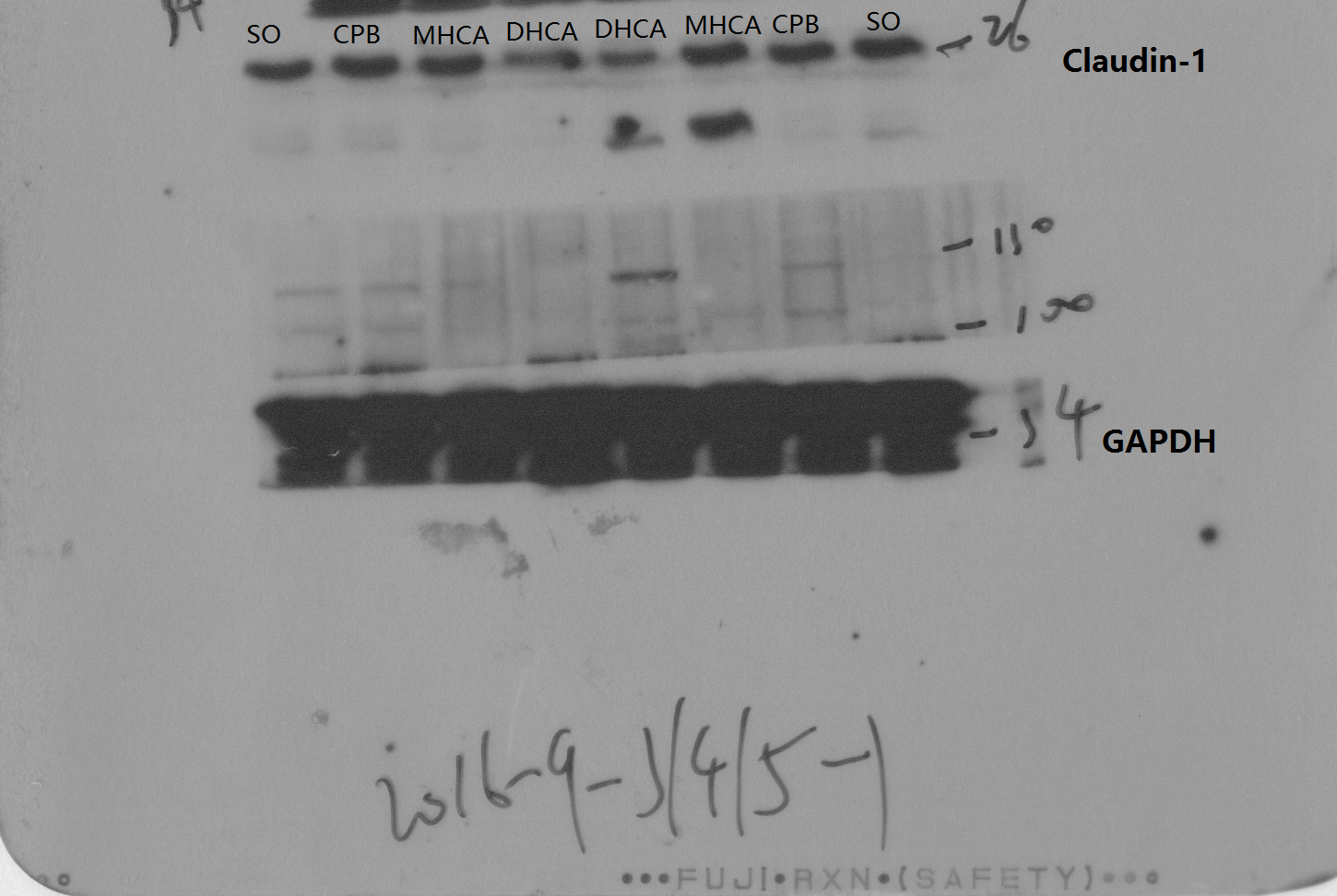

Supplement: S1 Fig — This figure shows the original uncropped blots of Claudin-1 at 180 min following reperfusion. SO, sham operation group; CPB, cardiopulmonary bypass group; DHCA, deep hypothermic circulatory arrest group; MHCA, moderate hypothermic circulatory arrest group. (TIF) [file pone.0163684.s001.tif]

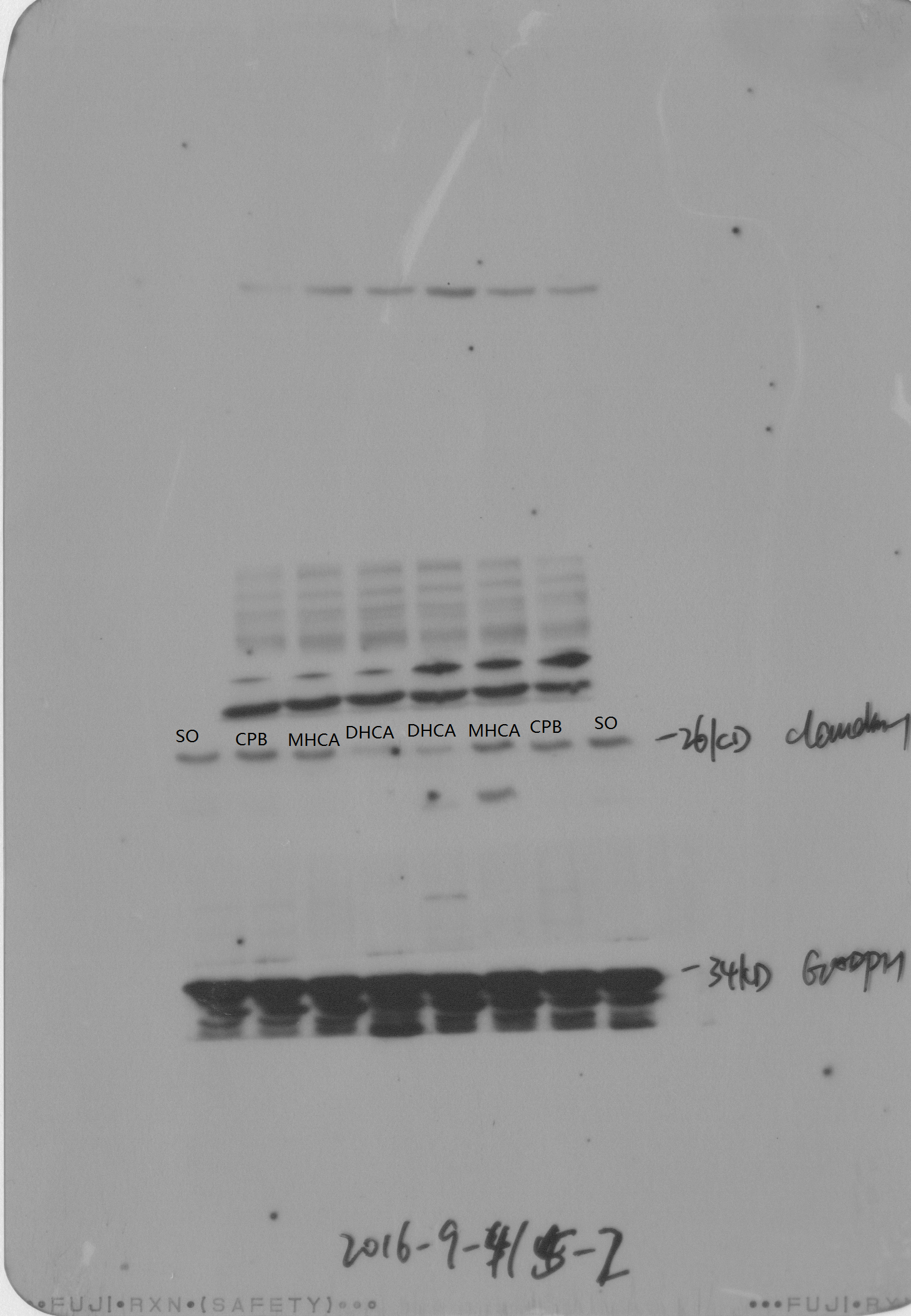

Supplement: S2 Fig — This figure shows the the original uncropped blots of Claudin-1 at 180 min following reperfusion. SO, sham operation group; CPB, cardiopulmonary bypass group; DHCA, deep hypothermic circulatory arrest group; MHCA, moderate hypothermic circulatory arrest group. (TIF) [file pone.0163684.s002.tif]

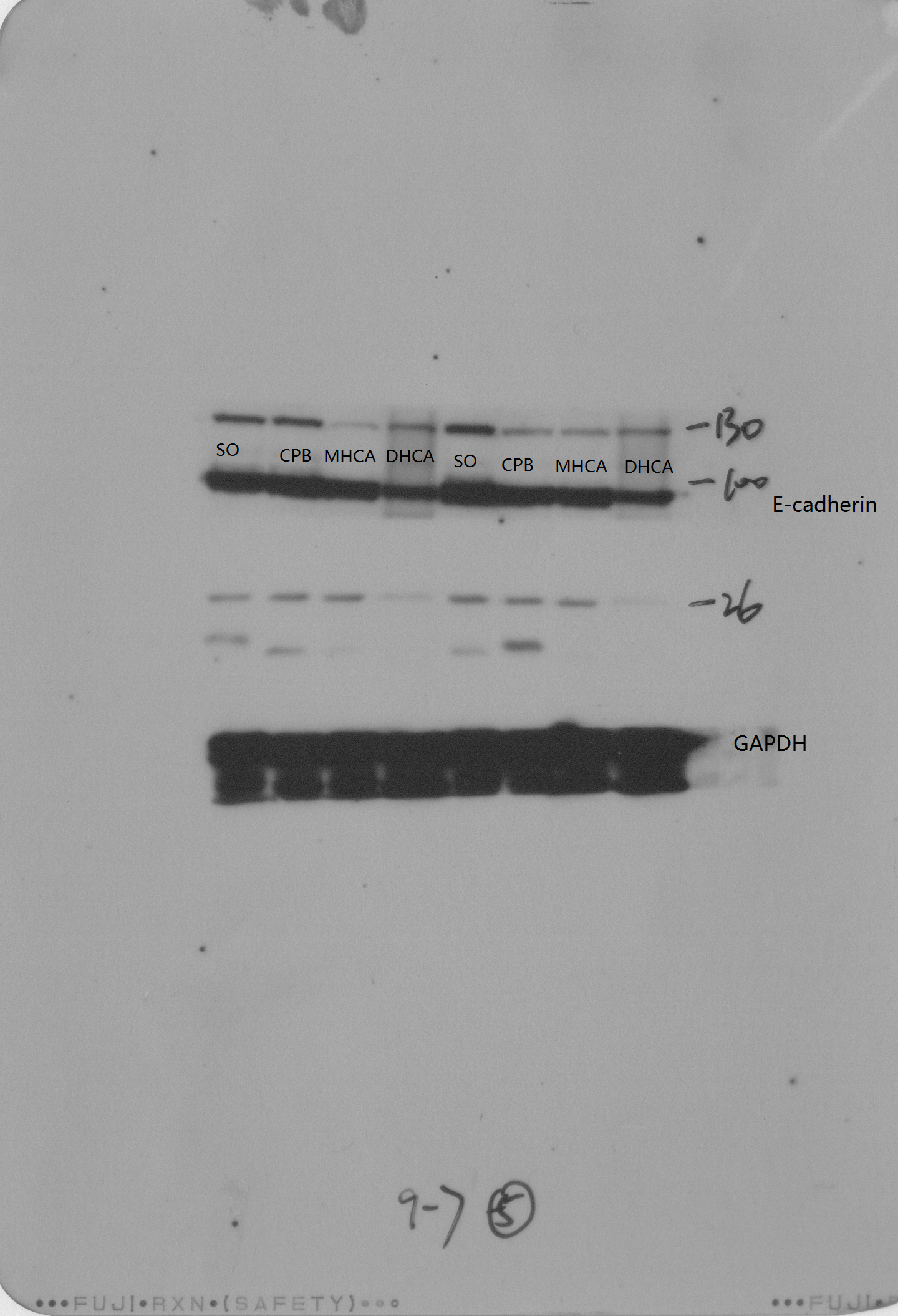

Supplement: S3 Fig — This figure shows the original uncropped blots of E-cadherin at 180 min following reperfusion. SO, sham operation group; CPB, cardiopulmonary bypass group; DHCA, deep hypothermic circulatory arrest group; MHCA, moderate hypothermic circulatory arrest group. (TIF) [file pone.0163684.s003.tif]

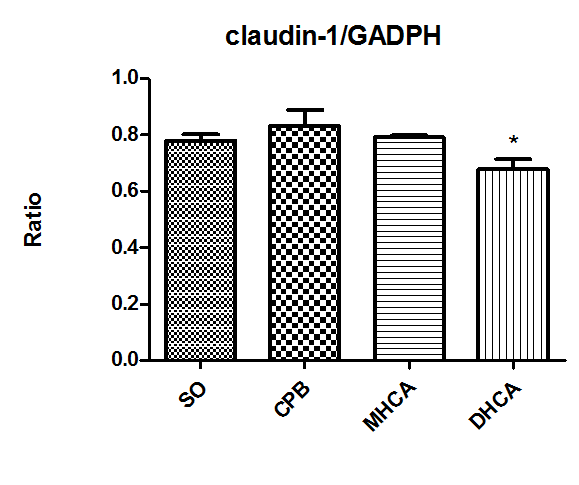

Supplement: S6 Fig — This figure shows the Claudin-1/GAPDH ratio of the groups. SO, sham operation group; CPB, cardiopulmonary bypass group; DHCA, deep hypothermic circulatory arrest group; MHCA, moderate hypothermic circulatory arrest group. *P < 0.05 DHCA vs. CPB (TIF) [file pone.0163684.s006.tif]

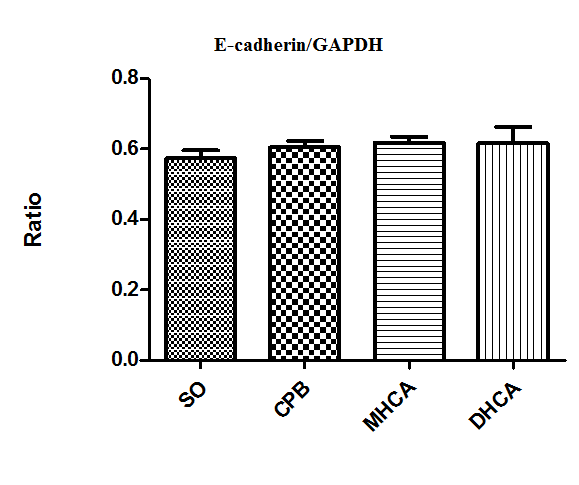

Supplement: S7 Fig — This figure shows the E-cadherin/GAPDH ratio of the groups. SO, sham operation group; CPB, cardiopulmonary bypass group; DHCA, deep hypothermic circulatory arrest group; MHCA, moderate hypothermic circulatory arrest group. (TIF) [file pone.0163684.s007.tif]
